# Characterization of T_1_ bias from lipids in MOLLI and SASHA pulse sequences

**DOI:** 10.1186/1532-429X-17-S1-W10

**Published:** 2015-02-03

**Authors:** Sarah B Thiesson, Richard B Thompson, Kelvin Chow

**Affiliations:** 1 Biomedical Engineering, University of Alberta, Edmonton, AB, Canada

## Background

Increased myocardial T_1_ values are associated with fibrosis and edema, while decreased values in Fabry disease have been attributed to the short T_1_ of infiltrative lipids [1,2]. The relationship between lipid concentration (LC) and best-fit T_1_ values is unknown. This study aims to determine the dependence of MOLLI and SASHA T_1_ values on LC.

MOLLI and SASHA[3] T_1_ mapping sequences are based on the bSSFP acquisition. bSSFP signal yield as a function of off-resonance frequency is well characterized [4,5], with phase inversion in sequential "bSSFP bands" and a profile shift as a function of resonance frequency, resulting in constructive/destructive interference between water and fat [6,7]. We hypothesized that lipids may decrease or increase T_1_ values as a function of off-resonance frequency.

## Methods

Bloch equation simulations of MOLLI and SASHA for 0:2:10% LC incorporated exact pulse sequence parameters including slice profiles and an accurate fat spectral line shape.

MOLLI and SASHA acquisitions (identical to simulations) were repeated 50 times, spanning 450 Hz of off-resonance (1.25 bSSFP bands) in both phantoms (LC of 0.5-10%), and in-vivo in three calf muscle regions with different LC [8]. *Acquisition Parameters:* 1.5T Siemens Sonata, single-shot bSSFP, 1.35/2.7ms TE/TR, 192x72 matrix, 360x270 mm FOV, 70° SASHA flip, 35° MOLLI flip [9]. T_1_ values were calculated using standard Look-Locker correction (MOLLI) or 2 and 3 parameter exponential models (SASHA) at each frequency increment across the bSSFP band.

## Results

**Figure 1:**
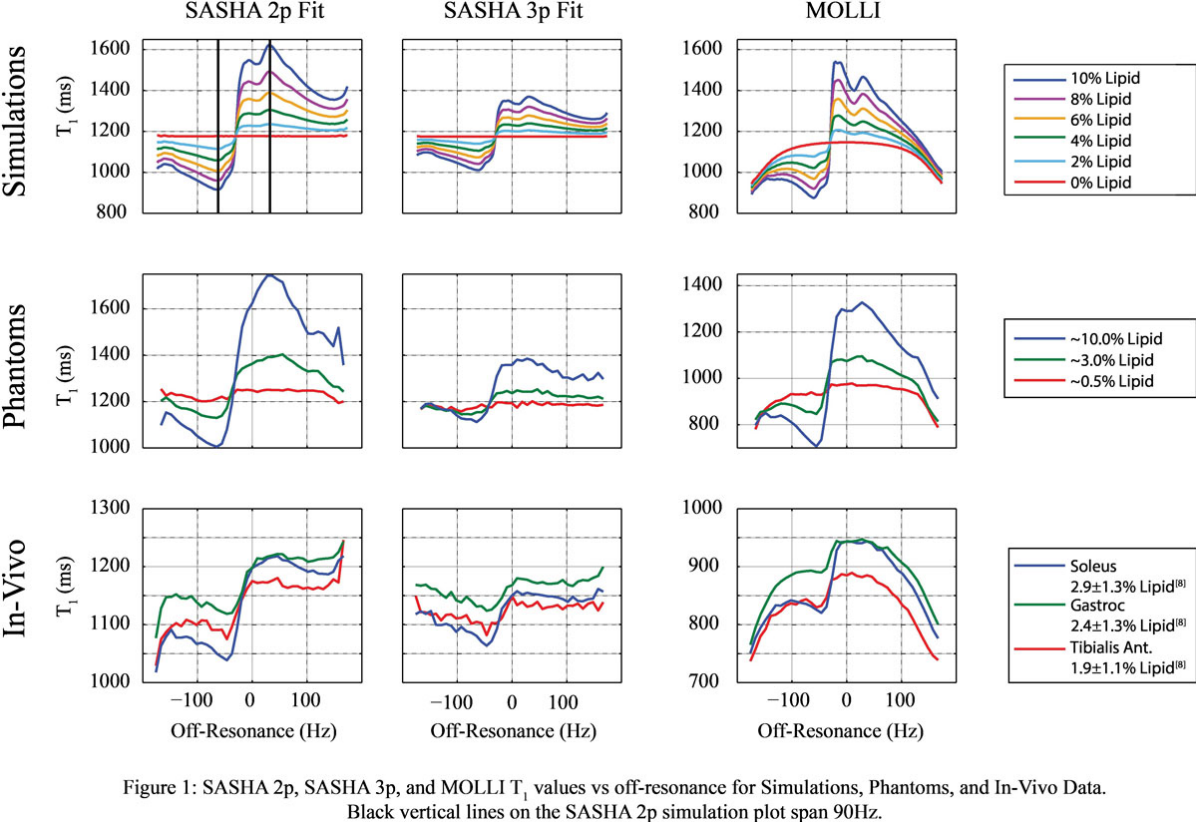
SASHA 2p, SASHA 3p, and MOLLI T_1_ values vs off-resonance for Simulations, Phantoms, and In-Vivo Data. Black vertical lines on the SASHA 2p simulation plot span 90Hz.

MOLLI and SASHA T_1_ values have an asymmetric relationship with off-resonance, with larger positive and negative biases with larger LC (Fig. 1). Over a small ±45 Hz range, a 1% LC gives rise to a T_1_ bias ranging from -39 to +26ms (MOLLI), -19 to +16ms (SASHA 3p) and -44 to +25ms (SASHA 2p). The location of the cross-over point is a function of field-strength and TR; these findings are specific to 1.5T and TR=2.7ms. MOLLI T_1_ values have an additional intrinsic dependence on off-resonance resulting in an underlying domed shape [10].

## Conclusions

Relatively low LC results in clinically relevant negative or positive shifts in tissue T_1_ over a narrow range of off-resonance frequencies with MOLLI and SASHA. Thus, increased or decreased native T_1_ values can potentially be ascribed to lipids, which can confound underlying increased water T_1_ values ascribed to fibrosis or edema and complicate the use of T_1_ mapping for indirect identification of lipids via reduced T_1_ values [1,2].
